# Development of a Novel Classification Approach for Cow Behavior Analysis Using Tracking Data and Unsupervised Machine Learning Techniques

**DOI:** 10.3390/s24134067

**Published:** 2024-06-22

**Authors:** Jiefei Liu, Derek W. Bailey, Huiping Cao, Tran Cao Son, Colin T. Tobin

**Affiliations:** 1 Department of Computer Science, New Mexico State University, Las Cruces, NM 88003, USA; hcao@nmsu.edu (H.C.); stran@nmsu.edu (T.C.S.); 2 Department of Animal and Range Sciences, New Mexico State University, Las Cruces, NM 88003, USA; 3 Carrington Research Extension Center, North Dakota State University, Carrington, ND 58421, USA; colin.tobin@ndsu.edu

**Keywords:** animal behavior identification, unsupervised machine learning, time series segmentation, clustering

## Abstract

Global Positioning Systems (GPSs) can collect tracking data to remotely monitor livestock well-being and pasture use. Supervised machine learning requires behavioral observations of monitored animals to identify changes in behavior, which is labor-intensive. Our goal was to identify animal behaviors automatically without using human observations. We designed a novel framework using unsupervised learning techniques. The framework contains two steps. The first step segments cattle tracking data using state-of-the-art time series segmentation algorithms, and the second step groups segments into clusters and then labels the clusters. To evaluate the applicability of our proposed framework, we utilized GPS tracking data collected from five cows in a 1096 ha rangeland pasture. Cow movement pathways were grouped into six behavior clusters based on velocity (m/min) and distance from water. Again, using velocity, these six clusters were classified into walking, grazing, and resting behaviors. The mean velocity for predicted walking and grazing and resting behavior was 44, 13 and 2 min/min, respectively, which is similar to other research. Predicted diurnal behavior patterns showed two primary grazing bouts during early morning and evening, like in other studies. Our study demonstrates that the proposed two-step framework can use unlabeled GPS tracking data to predict cattle behavior without human observations.

## 1. Introduction

Behavior patterns (e.g., grazing, resting, walking, and ruminating) of cattle provide critical information for livestock and rangeland management. Cattle have distinct diurnal activity patterns. Cattle graze primarily in the early morning and late afternoon and evening and often rest during midday and night [1]. The evaluation of cattle activity patterns can be used to monitor grazing behavior and indirectly forage intake [2]. The energy expenditure of cows is dependent on activity [3]. Cattle that walk farther and spend more time grazing as opposed the resting will expend more energy than cows that walk less and loaf longer. Cattle activity patterns are also useful for monitoring animal health and well-being [4,5]. Cattle are less active and move less when they become sick, which facilitates the detection of illness from the remote monitoring of animal activity using on-animal sensors [6,7]. Stress can also change cattle activity patterns. Cattle spend less time grazing and reduce feed intake during hot periods when they may be under heat stress [8]. The diurnal activity patterns of cattle and sheep changed when predators were present compared to patterns observed without predators [9,10].

Stockmen have traditionally monitored cattle health by observing their behavior, but this is time-consuming and sometimes impractical on extensive and rugged rangelands [11]. Manually observing changes in animal behavior requires much human effort as rangelands span a large spatial range and can include variable topography as well as shrubs and trees that interfere with visual observation. The development of on-animal sensors, such as ear tag accelerometers, can remotely monitor cattle activities and potentially provide that information in real time [12,13]. Video approaches have been used to record cattle behavior [14], but the large amount of associated data makes real-time transmission impractical for rangeland uses because of battery limitations. Accelerometers are also used to remotely monitor cattle behavior, but like video accelerometers, they produce large amounts of data recording three axes of movement at intervals of 10 to 20 Hz. To transmit data in real-time, accelerometer data are often condensed using edge computing techniques to provide indices that summarize data collected over 5-to-10-min intervals to a single value [13]. This summarization may limit its value for the detection of some behaviors. Tracking data typically produce less data than video and accelerometers, often recording positions at 1 to 15 min intervals, making real-time transmissions less of a burden on batteries [11].

Global Positioning System (GPS) devices can remotely record spatial movements. Tracking data can be used to monitor livestock health and well-being as well as the use of forage across their pastures [11,15]. For example, GPS tracking successfully detected simulated water system failures [16]. Statistical packages and analyses have been applied to evaluate tracking data and automatically utilize collected data from GPS devices [17] and other sensors [14,18]. More recently, machine learning (ML) and data mining techniques [19] are gaining interest due to their power to extract useful knowledge from large datasets.

Supervised machine learning techniques, such as random forests, support vector machines, and linear and quadratic discriminant analyses, have been used recently to predict livestock behavior from on-animal sensors and GPS tracking [17,20]. These supervised learning approaches require observations to train and validate machine learning models. However, it is time-consuming and expensive to manually collect visual observations, especially on extensive rangeland pastures.

Unsupervised learning is an additional machine learning technique. It can find patterns or group data without utilizing any user-labeled information (visual observations or video). Many machine learning tasks, such as feature selection, data segmentation, change point detection, anomaly detection, and clustering, can be solved using unsupervised learning techniques. Some unsupervised learning methods can generate intermediate data to facilitate the downstream machine learning tasks (e.g., classification [19,21]). As far as we are aware, no existing works have used state-of-the-art techniques, including time series segmentation, for analyzing GPS tracking data.

This study utilizes unsupervised learning approaches to assign livestock behaviors from GPS tracking data that span a wide spatial and temporal range with no human observation input. We design a new unsupervised learning framework to help categorize and label cow behaviors. The framework procedure partitions movement data into different segments using a state-of-the-art time series segmentation approach. This approach generates many segments, and the tracking data characteristics from each segment are similar. These segments are then categorized through clustering analysis into different groups, which is used to predict the cow’s behavior through time.

When we designed this framework, there were two major requirements. First, we expect the method to show good performance, including segmenting the data well, clustering the segments correctly, and running reasonably fast, all the while the predictions are consistent with the cattle expert’s expectations of typical cattle behavior patterns. The second requirement is that adopted algorithms have reasonably good interpretability. Conversely, two major technical challenges must be addressed when designing this framework. Most existing time series segmentation algorithms are too complicated to be utilized as the procedures may not be readily understood by biologists. We must choose an algorithm that provides good results and is explainable. Also, the length of the segments must be different. Clustering analysis algorithms must extract segments from equal-length sets from variable-length records.

This study integrates three unsupervised learning approaches, time series segmentation, feature extraction, and clustering, into GPS tracking data and then presents and evaluates the corresponding predictions.

## 2. Methodology

We designed a two-step framework for the analyses based on observations and livestock behavior knowledge. When a cow displays one behavior, that behavior may persist for an extended time period. For example, cows often lie down for an hour or more, which means that the remotely sensed features should show similar values for a continuous period of time for one behavior. The recorded feature values for a cow should be similar when the cow is exhibiting the same behavior. For example, a cow’s velocity (or rate) is often similar if it is walking despite the change in location.

**Figure 1:**
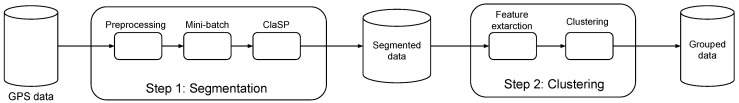
The proposed two-step analysis framework.

The two-step framework works as follows (Figure 1). The first step is to partition the long sequence of recorded positions (a cow’s movement path) into multiple shorter non-overlapping segments. In each segment, a cow’s behavior should be the same. These segments may have different lengths and belong to different behaviors or share the same behavior. The second step is to group the segments sharing similar characteristics into different clusters, where each cluster represents one behavior or one behavioral bout.

### 2.1. Dataset Description

Tracking data were collected as part of another study completed at Deep Well Ranch located near Prescott, Arizona [16]. The study site is a 1096 ha pasture with gentle undulating terrain $(112^{\circ }29^{\prime }$ W, $34^{\circ }41^{\prime }$ N). The climate at the site is classified as cold semi-arid (Bsk) according to the Koppen–Geiger Climate Zone [22].

A total of 120 Corriente cows and their calves grazed the study pasture. Cows varied in age from 2 to 15 years. These cows were raised at Deep Well Ranch and were familiar with the study pasture. Eight of the cows in the herd (randomly selected) were tracked with IgotU GT-120 or IgotU GT 600 GPS receivers (Mobile Action Technology Inc., Taipei, Taiwan) mounted on collars [23]. Cows were tracked at 2 min intervals from 28 May 2018 to 22 June 2018 (i.e., a total of 26 days).

**Figure 2:**
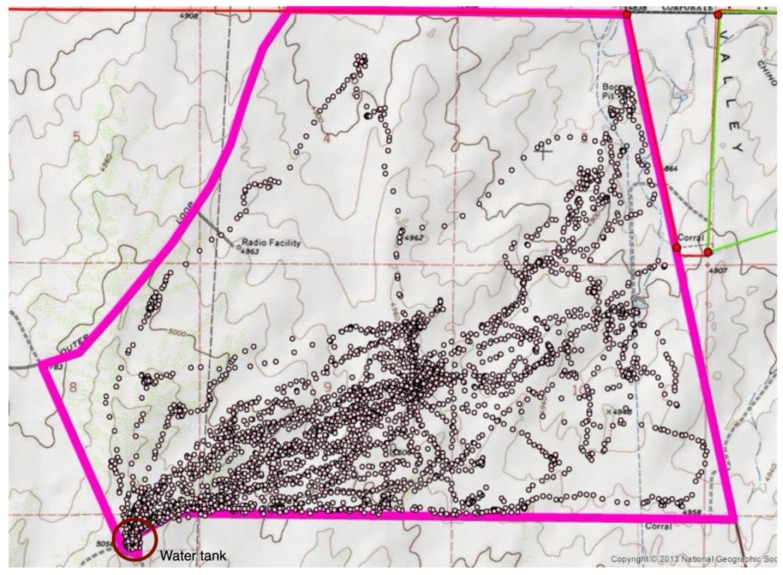
The recorded GPS positions of cow 225 show the trajectory (or pathway) of her movements from 28 May to 22 June. Positions are small circles and were recorded at 2 min intervals using a GPS collar. The pink lines represent the boundary of the 1096 ha rangeland pasture. Water was available to the cows in the southwest corner of the pasture (indicated by a red circle).

Three features (the course, rate, and distance from water) were selected from the GPS tracking data for analysis. The course is the direction in which a cow moves between recorded positions and was calculated by the GPS device. The rate was calculated by dividing the distance between two consecutive positions by the elapsed time between the position recordings. The “distance from water” feature is the Euclidean distance from the recorded position to the only water source located in the southwest corner of the pasture (Figure 2).

### 2.2. Terminology

This section defines the terminology used in this paper.

**Definition** **1** (Record). *A record of a dataset keeps the information of an object or the status of an object at different times or different locations.*

A record is also called a sample or an instance. This paper uses record, sample, and instance interchangeably. In our study, a record/sample is the cow’s movement information at a timestamp.

**Definition** **2** (Feature). *A feature (denoted as f) describes the intrinsic characteristic or property of a sample.*

Examples of features include a cow’s location, movement velocity, and movement direction at the recording time.

**Definition** **3** (Time series/sequence). *A time series (or sequence) S is an ordered list of records $\left[\left(t_{1},r_{1}\right),\left(t_{2},r_{2}\right),\ldots ,\left(t_{n},r_{n}\right)\right]$ where $t_{i}<t_{j}$ when i<j and $r_{i}$ is the record at timestamp $t_{i}$.*

The tabular time series data represent the movement of a cow. Each row represents a record consisting of recorded values for multiple features at a specific time. The length of a time series is the number of records in it. Given $S=[\left(t_{1},r_{1}\right),\left(t_{2},r_{2}\right),\ldots ,\left(t_{10},r_{10}\right)]$, the length is 10.

**Definition** **4** (Segment/subsequence). *A segment of a sequence S is a subsequence with records (sequential recorded cow locations) in a continuous time period. One segment Sij can represent the subsequence $[\left(t_{i},r_{i}\right),\left(t_{i+1},r_{i+1}\right),\ldots ,\left(t_{j-1},r_{j-1},\left(t_{j},r_{j}\right)\right]$, where 1≤i≤j≤ length of S and the length of $S_{ij}$ is j−i+1.*

### 2.3. Step 1: Segment Sequences of Cow Movement Using ClaSP Approach

Step 1 partitions the movement sequence of a cow into different segments using a time series segmentation algorithm such that the behaviors of the cow in one segment are similar.

We choose to use a state-of-the-art time series segmentation approach called ClaSP [24] to partition the movement data after investigating the many existing time series algorithms. The ClaSP method was chosen based on two factors. First, it is a state-of-the-art method proposed in 2021 [24]. Second, it utilizes a very easy-to-understand K-nearest neighbor (KNN) classifier as a building block and applies self-supervision [25]. Thus, results from this method are easy to understand and explain.

#### 2.3.1. The ClaSP Algorithm

For a given sequence $S=[\left(t_{1},r_{1}\right),\left(t_{2},r_{2}\right),\ldots ,\left(t_{n},r_{n}\right)]$, ClaSP uses a divide and conquer solution [26] by recursively splitting a long segment into two smaller segments until it obtains a predefined number of segments (which is a parameter).

This algorithm utilizes another parameter *w* as the length of a sliding window to extract subsequences. For each time point $t_{i}$, a subsequence/segment $S_{i,i+w-1}=\left[\left(t_{i},r_{i}\right),\left(t_{i+1},r_{i+1}\right),\ldots ,\left(t_{i+w-1},r_{i+w-1}\right)\right]$ of length *w* is extracted. In total, for a length−n sequence, n−w+1 segments can be extracted, and let Σ be the set of these segments. For each timestamp $t_{i}$, the algorithm computes a score $\sigma_{i}$ to estimate whether we can split the segment at $t_{i}$. The higher the score $\sigma_{i}$ is, the more reason to partition *S* at timestamp $t_{i}$.

The score $\sigma_{i}$ (for splitting at $t_{i}$) is calculated as follows.

- For $t_{i}$, the algorithm first labels all the length−w segments with a class label (either 0 or 1). All the segments in Σ before $t_{i}$ are labeled with one class label (e.g., label 0), indicating they belong to one behavior. All the segments in Σ that correspond to or after $t_{i}$ are labeled with another class label (e.g., label 1), indicating a different behavior. These class labels are considered as ground truth behavior labels for splitting at $t_{i}$.
- For each segment in Σ, the algorithm finds *K* segments from Σ that are most similar to it. These *K* segments are called the *K* nearest neighbors (KNNs) of $S_{i,j+w-1}$. The predicted class label of the subsequence $S_{i,j+w-1}$ is the majority class label of its KNNs.
- Based on the grand truth labels (Step 1) and the predicted labels (Step 2), the algorithm calculates a validation score, the Area Under Curve (AUC) score. This score is $\sigma_{i}$.

The time point with the highest splitting score $\sigma_{i}$ is treated as a splitting point if it is higher than a threshold to generate two segments (one segment $S_{1,i-1}$ before $t_{i}$ and one segment $S_{i,n}$ after $t_{i}$).

If more segments need to be generated, the above algorithm is recursively applied to the two segments $S_{1,i-1}$ and $S_{i,n}$. Such a process can be recursively applied to any segment to conduct further splitting until no segment can be split (which means that the highest splitting score is lower than the threshold).

There are two important parameters in this method. One is the sliding window size *w*. Another one is the splitting score threshold. Setting the parameters is not straightforward. According to [24], the sliding window size needs to be appropriately decided by, in this particular case, livestock experts. If the sliding window size is not properly set, the algorithm either detects many false splitting points (when *w* is too small) or misses segments (when *w* is set too large). Setting the splitting score threshold is also crucial so that the algorithm can stop at a proper stage. In this paper, we manually analyzed single-day time series segments and estimated the daily number of change points.

#### 2.3.2. Data Preprocessing and Mini-Batch Creation

Despite the fact that the ClaSP is a state-of-the-art approach, it cannot be directly utilized because of its constraints. It only works for univariate time series data. I.e., in our definition of *S*, $r_{i}$ is just a scalar value, instead of an instance with values for multiple features. Our dataset is a multivariate time series because the record at each timestamp contains values for multiple features. This setting affects how we calculate the distance between subsequences. To address this issue, we utilize a very simple strategy. For each length−w subsequence, we concatenate the subsequences of all the selected features to form one subsequence.

Another major issue we encountered when applying the ClaSP algorithm is that no meaningful segments can be found from the entire tracking sequence for a cow (in this case, 26 days), even though we tried different parameter values. Through the process, we find that the issue is caused by the long sequence (over 20,000 locations per cow). The original algorithm never used the long sequences that were used in our study. To address this issue, we propose a mini-batch idea.

The “mini-batch idea” is based on an observation. When we find KNNs for a segment, we do not need to look at all the movement trajectories (for 26 days). Instead, the movement in a shorter period that is close to the segment (e.g., within one day) can better help identify the segments with similar behavior. The mini-batch idea works as follows. When calculating the KNNs for each segment, we only use the segments within a small time window. With the domain expert’s help, we set the small batch size to 120 (4 h). With this small batch, the experiments show (Section 3.2) that we can easily set window sizes as 1, 2, or 3. Utilizing this mini-batch, the calculation of KNNs improves because the number of candidates for the KNN calculation decreases dramatically (from the whole long sequence to the subsequence in a shorter period).

### 2.4. Step 2: Clustering Analysis

This step aims to group the segments into different clusters such that the segments in one cluster exhibit similar behavior. In this clustering analysis, one segment is one clustering instance. Note that each segment contains data from three features ($f_{r}$, $f_{c}$, $f_{d2w}$), and the segments’ lengths may vary.

For clustering algorithms to work, all the instances for clustering (segments) need to have the same number of features. However, the segments we obtained from the previous step have different lengths. We first need to extract features from each segment. In our study, we used the mean and standard deviation of the original data features (the rate, course, and distance to water features). After extensive experiments, we found that using only the statistical features from the rate feature resulted in the most consistent clusters. Thus, our reported clustering results were based only on the rate feature.

#### Segment Clustering

We choose to use the hierarchical clustering approach in our analysis because hierarchical clustering analysis does not require any input parameters and allows users to form and visualize different clusters.

**Figure 3:**
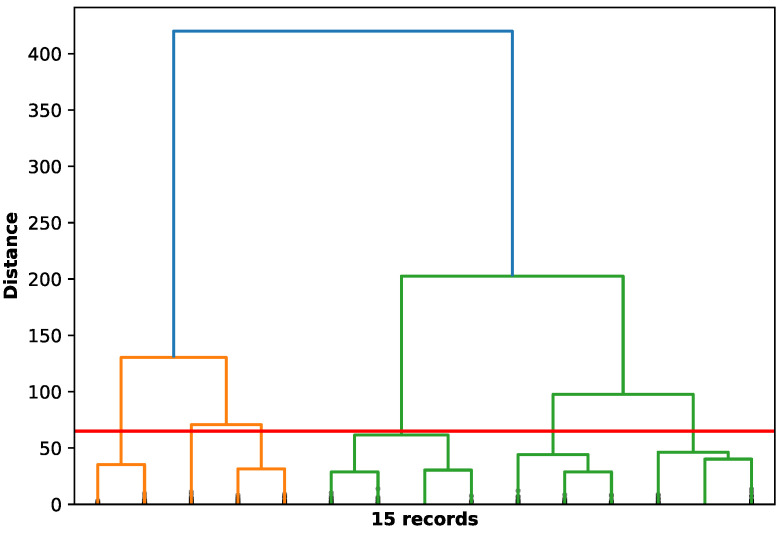
Dendrogram obtained using hierarchical clustering. The horizontal red line is the selected threshold for cow 225. Different colors are used to represent different hierarchical clusters.

We apply hierarchical clustering to group segments based on the features we created for each segment. The hierarchical clustering creates a dendrogram to clearly show how the instances (i.e., segments) are grouped together step by step to form different groups/clusters (Figure 3). Many rangeland studies that classified cattle behavior from remotely collected monitoring data (typically GPS tracking and accelerometers) predicted resting, grazing, and walking [27,28]. We also hypothesized that cattle behavior may differ when cattle are near water (less than 200 m) compared to far from water. These three behaviors (grazing, resting, and walking) and whether the cow is near or far from water create six situations. Correspondingly, we set the number of clusters to six.

## 3. Case Study

All the methods were implemented using Python 3.9 and tested on a Mac with an M1 chip. The Scikit-learn (version 1.0.2) library was used to preprocess the data and build the clustering model. The ClaSP code is downloaded from [24] and modified to reflect changes described in Section 2.3.1.

### 3.1. Data Cleaning

We removed three of the eight tracked cows from the dataset because the tracking data were incomplete. The GPS failed to record positions for several hours or even days. The tracking data for the other five cows had minimal missing data and were used for our experiments.

### 3.2. Model Parameter Tunning

Proper parameter values need to be configured to make the algorithm work. We choose parameters by extensively analyzing the data of one cow’s trajectory on one day to determine the effect of the window size on the ClaSP segmentation algorithm.

**Figure 4:**
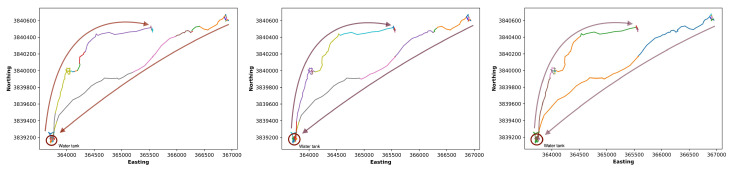
Effect of window sizes (1, 2, and 3) on segmentation for cow 225 on 20 June 2018. The map on the left uses a window size of 1 for segmentation, and the middle and right maps show segmentation with window sizes of 2 and 3, respectively. (Color changes within a map reflect different segments. Fewer segments were assigned to this portion of the trajectory with larger window sizes (1 vs. 2 vs. 3).

We conducted experiments to examine the effect of different parameter settings on the segmentation algorithm. The first parameter we tested was window size w. Our results (Figure 4) show that the algorithm with smaller window sizes generates more (but false positive) segments, while the algorithm misses important segments when using larger window sizes. Comparing three window sizes 1 and 2 (Figure 4), we can observe that the ClaSP method found many segments. For example, the algorithm partitions the movement close to the location (3,643,000, 3,840,000) into two segments highlighted in navy and orange. However, these two segments do not have intrinsic differences because the cow constantly moves while keeping the same direction. Thus, these two segments should be combined as one segment.

The right map of Figure 4 shows the segmentation results with a larger window size 3. The ClaSP method misses segments for this window size. For example, in the trajectory between locations (365,700, 3,840,150) and (366,900, 3,840,600), the cow’s speed and direction change many times. However, the method did not detect them.

Based on the above analyses, we chose the following parameter setting for our framework. For the ClaSP algorithm, we set three parameters: the window size w of ClaSP (Section 2.3.1) was set to be two so that each sliding window can cover movement in 4 min; the splitting score threshold (i.e., AUC score) (Section 2.3.1) was set to 70% as the filtering condition of the ClaSP algorithm; and the mini-batch size was set to 120, representing splitting the data into 4 h subsequences (Section 2.3.2). For the clustering method, we set the number of clusters to six (Section 2.4).

## 4. Results and Discussion

### 4.1. Results of Segmentation

**Figure 5:**
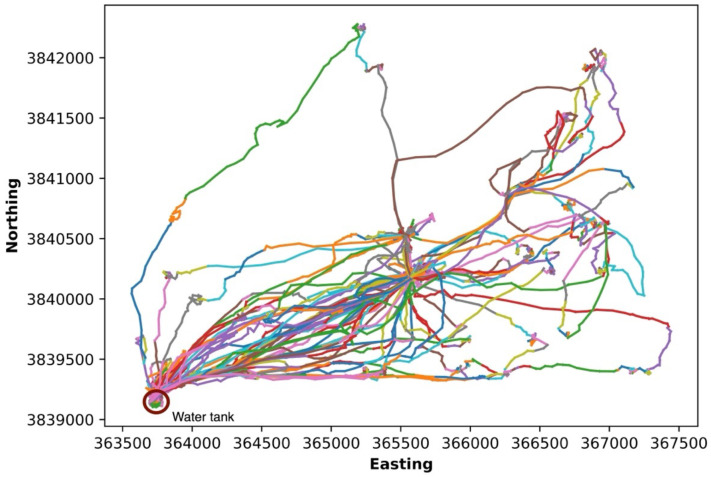
Segmentation of the trajectory or path of cow 225 from 28 May 2018 to 22 June 2018. Color changes along the path represent the different segments. For example, the green segment in the upper left of the map is one segment. The adjacent orange segment (shorter and more sinuous) is a separate segment.

The segments changed in correspondence to changes in the movement patterns (Figure 5). For example, positions were recorded apart in a straight line (a change in color in Figure 5) from positions located near each other in a group. This demonstrates that our segmentation strategy can appropriately partition the cow trajectory utilizing three features (the rate, course, and distance to water) without any user input or prior knowledge. The differentiation among segments (Figure 5) demonstrates that the algorithm has identified segments in the trajectory that indicate changes in direction, which often occur during grazing [29]. Another example is that the red and orange segments (i.e., the segment change) on the top right of the trajectory show a clear direction change in the cow’s movement. On the other hand, the cow’s trajectory at location (367,000, 3,840,500) shows grouped position points with constant direction changes, indicating that the cow is not moving; the direction changes likely reflect GPS error and suggest the cow may be resting (standing or lying). Other features are also useful in helping generate the segments. For example, ClaSP can detect and segment the cow’s moving path near the water tank based on the distance-to-water feature, which is reflected by the different segments near the water tank.

### 4.2. Discussions of Segment Clustering Results

**Figure 6:**
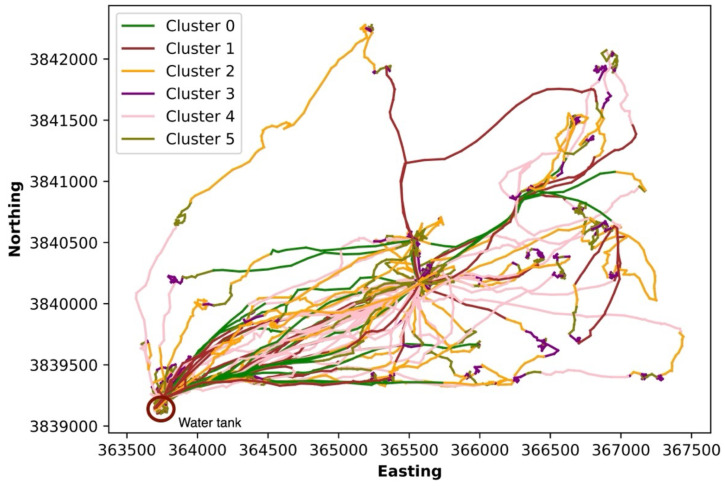
Map of clusters of the trajectory (path) cow 225 from 28 May 2018 to 22 June 2018. Different colors represent different clusters. Clusters are combinations of consecutive segments (e.g., Figure 5) with similar properties.

Six different clusters were created by aggregating segments using the rate feature (Figure 6). In contrast to our expectations, the distance to water was not useful for clustering. Six different colors represent six different clusters with 26 days of data from one cow (Figure 6). For example, the segments belonging to the pink cluster (Figure 6 Cluster 4) are aggregated, indicating that the cow stayed at the same location, and direction changes are likely from GPS error. Intuitively, this cluster indicates that the cow is resting. The olive (Figure 6 Cluster 5), crimson (Figure 6 Cluster 1), and purple (Figure 6 Cluster 3) clusters represent successive positions relatively long distances apart along the same general course, which suggests walking behavior. The green (Figure 6 Cluster 0) and orange (Figure 6 Cluster 2) clusters are tortuous while the cow moves and switches directions, which might indicate grazing. These clusters represent typical behavioral activities of cattle and could be used to monitor their health and well-being. For example, cattle that rest and limit their grazing during the early morning and evening (normal grazing bouts) may be ill. Tobin et al. [30] found a distinct change in the diurnal behavior pattern of grazing when cattle became ill with bovine ephemeral fever. Grazing activity markedly declined at the onset of their illness, which was especially noticeable in the morning and evening. Identifying these clusters and the associated behavior (i.e., labeling, see below) facilitates the remote monitoring of cattle grazing rangelands and the potential detection of illness and other welfare concerns [5,11].

### 4.3. Classifying/Labeling Clusters with Behaviors

**Figure 7:**
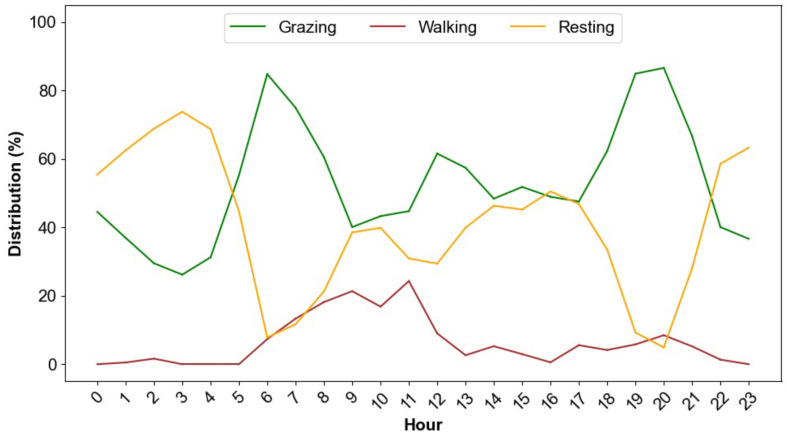
Diurnal distribution of activities by cow 225. Activities were categorized into three labels, grazing, resting, and walking. The distribution reflects hourly averages across the entire tracking period 28 May to 22 June 2018.

We labeled/classified each cluster with a cow behavior based on (i) the mean, median, and standard deviation of the rate for each cluster (Table 1) and (ii) the diurnal pattern (hourly) of a cow’s clusters (Figure 7). Intuitively, when $S_{r}<4.5$ m/min ($S_{r}$ represents the mean rate of a cluster across all cows), the cow is resting; when 4.5 m/min $<S_{r}<25$ m/min, the cow is grazing; and when $S_{r}>25$ m/min, the cow is walking, and this intuition is consistent with the criteria used by Augustine et al. [28] Nyamuryekung’e et al. [31] and Tobin et al. [16] used the following values for resting (rate <2.34 m/min), grazing (2.34< rate <25 m/min) and walking (rate >25 m/min) (Table 2). Ungar et al. [27] reported average cattle grazing velocities of 5 to 6 m/min and walking velocities of 30 m/min. The diurnal activity pattern (by the hour) using our clusters (Figure 7) is also consistent with the findings of Walker et al. [32] and Gregorini Pablo [33], where the cows’ common daily activities are 38∼48% grazing, 50∼57% resting, and 2∼5% walking.

**Figure 8:**
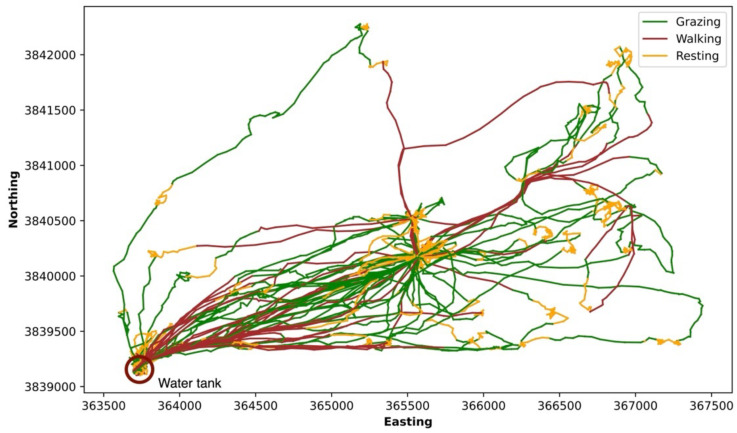
Map of predicted behaviors of cow 225 from 05/28/2018 to 06/22/2018. Clusters were combined into predicted behaviors (grazing, resting, and walking). Colors represent the predicted behaviors.

**Figure 9:**
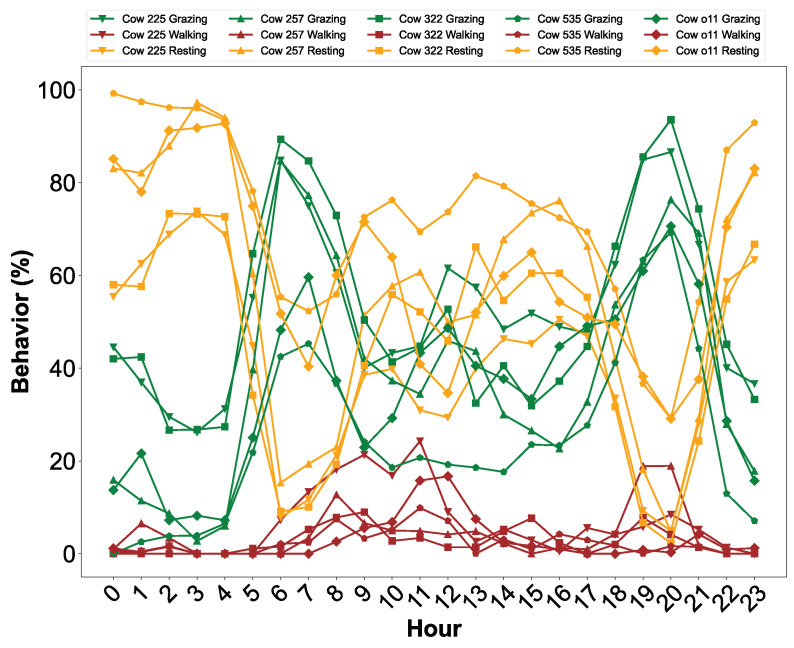
Diurnal distribution (hourly) of predicted behaviors of all the cows. The diurnal distribution reflects hourly averages of three behaviors (grazing, resting, and walking) for each cow across the entire tracking period (May 28 to 22 June 2018).

We plotted the trajectory of cow 225 into three groups, grazing, walking, and resting (Figure 8). The yellow line represents resting, and the corresponding cow trajectory looks like the cow’s movement remains close to a specific location on the plot. The red line represents the cow walking, which is a small portion of the whole trajectory. When the cow is walking, it often moves directly to and from water. The walking trajectory shown in red is relatively straight and provides evidence that the cow is walking. Finally, the winding green line represents grazing. Larson-Praplan et al. [29] measured the tortuosity of cattle movement paths and found that turning angles varied in response to forage characteristics of patches. Cattle turned frequently while grazing and often modified turning angles and increased tortuosity to remain in preferred patches (typically 30 to 90 m in size). The diurnal pattern is also consistent with normal cow behavior with low levels of activity at night and active periods during the early morning and evening (typical times for grazing bouts). The timing of predicted walking bouts is also consistent with the expectation of cattle walking to water during the late morning [34,35,36]. The diurnal pattern of our example cow (#225) is similar to the patterns of the other four cows (five total) evaluated (Figure 9 and Table 2). The similarity of the activity patterns among the five cows evaluated shows that the proposed two-step non-supervised machine learning approach should work for multiple cows.

#### Discussions of the Soundness of Labeled Clusters

We studied five cows over 26 days, plotting their hourly activities. From 10 p.m. to 6 a.m., they rest more than grazing, with no walking. By 6 a.m., they wake, begin the grazing bout, and start walking. The peak walking time is around noon, possibly indicating movement toward water sources. A second grazing bout was predicted during 5 to 8 p.m. In the summer when our study occurred, cattle typically have two primary grazing bouts (early morning and evening [29,33]). Cattle may avoid grazing during midday and instead travel to water and rest to help compensate for high temperatures.

Our framework can segment a cow’s trajectory and group movements exhibiting similar behaviors. Similarly, we demonstrate the reasonability of our clustering and labeling results by examining cows’ average daily behavior patterns. This framework can aid researchers in identifying animal behaviors without having to observe cows in extensive and/or rugged rangeland pastures to use in supervised machine learning models. These observations are very labor-intensive. For example, Augustine and Derner [28] observed cattle for a total of 504 h to use in supervised machine learning analyses with regression trees. In other research that used supervised machine learning to classify behavior, two observers monitored cows during daylight hours for 9 days in one study and three observers recorded cattle behavior for 8 days in another study [27]. Most machine learning approaches used to classify cattle behavior are more accurate if the model is developed separately for each cow rather than pooling all cows together in a generic model [37]. This requires many observations of each cow, which is difficult in extensive and rugged rangeland. This unsupervised machine learning approach can predict the behavior of individual cows from GPS tracking data without the time and expense of collecting observations to train the model. As far as we know, this is the only study using unsupervised machine learning to classify cattle behavior from tracking data. Unsupervised machine learning has been used to predict and evaluate cattle behavior in the dairy industry using accelerometer and milking order data [38,39]. Unsupervised machine learning models and accelerometer data from dairy cattle were also used to predict estrus [40,41].

## 5. Conclusions and Future Work

In this study, we designed an unsupervised machine learning framework to classify cow behaviors based on their movements. The framework first partitions the movement data (represented as a long sequence) into shorter segments and then clusters the segments into different groups. Each group represents one behavior. The approach was applied to a dataset acquired from five cows in one month. The pattern of cattle’s behaviors was consistent with previous studies. Our research suggests that animal behavior can be classified into different behaviors using GPS tracking data without observational data to train models, saving time and labor. With the development of real-time tracking technologies, unsupervised machine learning could be a valuable tool to help monitor livestock behavior on an individual animal and site-specific basis.

In the future, we will create a pipeline to conduct this unsupervised analysis so that domain experts, in this case, those studying livestock behavior, can directly utilize this framework to label (classify) cow behaviors and apply them to the other datasets. Another direction of future work is to design an online analysis approach or incremental method to segment cow movement. The proposed two-step framework works offline. The offline approach cannot be utilized to analyze movement data in real time. This technique has advantages over using specific velocity values for assigning behavior (e.g., [31]), because it is derived from the collected data of individuals and will vary among and across herds. Potentially, this unsupervised machine learning framework may increase the availability of predicted behavior patterns from remotely collected GPS tracking data that are reflective of the unique characteristics of each animal and each location.

Most importantly, the results of this unsupervised machine learning algorithm must be validated with actual observations. Although the purpose of this proposed framework is to avoid collecting visual observations, a study using observations to validate the accuracy of this approach is needed. In addition, specific studies are needed to compare the efficacy of supervised and unsupervised machine learning approaches for predicting cattle behavior patterns from remotely collected tracking data.

## Figures and Tables

**Table 1:** Mean rate, standard deviation, and median of the six clusters and associated predicted behaviors for cow 225.

Cow 225 Clusters	Rate Mean	Rate Standard Deviation	Rate Median	Label
Cluster 0	46.64	25.04	52.61	Walking
Cluster 1	24.84	26.73	19.15	Walking
Cluster 2	6.76	12.51	0.00	Grazing
Cluster 3	1.09	3.72	0.00	Resting
Cluster 4	13.18	19.86	0.00	Grazing
Cluster 5	3.56	7.52	0.00	Resting

**Table 2:** Average rate (m/min) of predicted walking, grazing and resting behaviors for all cows.

	Cow Identification					
Activities (m/mins)	225	257	322	535	o11	Overall Average
Walking	35.74	38.38	39.83	61.18	47.45	44.09
Grazing	9.97	12.20	12.07	16.39	13.86	12.90
Resting	2.33	2.42	1.31	2.16	1.98	2.11

## Data Availability

The cattle tracking data supporting this study were summarized and first published by Tobin et al. (2021), which is cited below [16]. Requests from the cattle tracking data collected at Deep Well Ranch and used in this study must be directed to the authors and approved by New Mexico State University and Deep Well Ranch.
